# Impact on learning of an e-learning module on leukaemia: a randomised controlled trial

**DOI:** 10.1186/1472-6920-12-36

**Published:** 2012-05-28

**Authors:** Yuri Morgulis, Rakesh K Kumar, Robert Lindeman, Gary M Velan

**Affiliations:** 1 Department of Pathology, School of Medical Sciences, Faculty of Medicine, The University of New South Wales, Sydney, NSW, 2052, Australia; 2 Department of Haematology, Prince of Wales Hospital, Sydney, NSW, 2031, Australia

**Keywords:** E-learning, Computer-assisted learning, Medical education, Leukaemia

## Abstract

**Background:**

e-learning resources may be beneficial for complex or conceptually difficult topics. Leukaemia is one such topic, yet there are no reports on the efficacy of e-learning for leukaemia. This study compared the learning impact on senior medical students of a purpose-built e-learning module on leukaemia, compared with existing online resources.

**Methods:**

A randomised controlled trial was performed utilising volunteer senior medical students. Participants were randomly allocated to Study and Control groups. Following a pre-test on leukaemia administered to both groups, the Study group was provided with access to the new e-learning module, while the Control group was directed to existing online resources. A post-test and an evaluation questionnaire were administered to both groups at the end of the trial period.

**Results:**

Study and Control groups were equivalent in gender distribution, mean academic ability, pre-test performance and time studying leukaemia during the trial. The Study group performed significantly better than the Control group in the post-test, in which the group to which the students had been allocated was the only significant predictor of performance. The Study group’s evaluation of the module was overwhelmingly positive.

**Conclusions:**

A targeted e-learning module on leukaemia had a significant effect on learning in this cohort, compared with existing online resources. We believe that the interactivity, dialogic feedback and integration with the curriculum offered by the e-learning module contributed to its impact. This has implications for e-learning design in medicine and other disciplines.

## Background

e-learning is evolving in parallel with technological advances, and as a result is able to create more authentic learning experiences. e-learning resources have the potential to contribute to the goals of higher education, by supporting autonomous, life-long, student-centred learning. This can be achieved by creating a platform that is malleable for different types of learners and different ability levels while maintaining the same standard of information and accessibility for students and teachers [1].

In recent years, e-learning has become increasingly integrated into mainstream medical education, due to greater levels of acceptance by staff and increased expectations by students. As a consequence, medical students currently have access to an abundance of e-learning resources, and are often paralysed by the overwhelming amount of information available [2]. Furthermore, most such information is either too general or too detailed for the purposes of their studies. In contrast, e-learning that is appropriately structured and focused on topics known to be conceptually difficult [3] may potentially be of significant value to students.

In general, there is a dearth of reliable data regarding the efficacy of e-learning, as well as a lack of funding available to support long-term studies that monitor and evaluate the ongoing impact of e-learning innovations [4,5]. This is compounded by highly variable methodology between studies, as well as the lack of widely accepted metrics to evaluate e-learning resources. All of this is exacerbated by the ethical difficulties of running randomised controlled trials to establish the efficacy of e-learning. Nevertheless, existing studies of e-learning for medical students in a variety of disciplines, such as those in anatomy [6], paediatrics [7,8] and pathology [9], indicate that e-learning can have significant benefits.

Importantly, a meta-analysis of studies comparing e-learning with face-to-face teaching revealed that although e-learning is significantly better than no intervention, in most cases it is equivalent to ‘traditional’ teaching methods [10]. The logical extension of this finding is that research should now focus on comparing the efficacy of differing forms of e-learning, to determine the modes and contexts in which e-learning might be most useful [11].

Adequate integration of e-learning technology into the curriculum is an important factor in perceived efficacy for both students and teachers [12,13]. Integration requires that an e-learning module has explicit learning objectives, which provide a high level of relevance and validity [14]. In addition, Wong and colleagues [15] suggest that e-learning is most engaging for students when it places learning in context, as well as drawing on prior learning.

Based on the above, we hypothesised that effective e-learning in medical education might be best achieved via curriculum-based modules that bring diverse concepts together in an authentic clinical context, while emphasising interactivity and feedback, as well as integrating with, and expanding on, prior learning.

At the University of New South Wales (UNSW), the six-year undergraduate medicine program is structured around the development of core graduate capabilities, which are considered fundamental for successful practice directly following graduation and throughout a career in medicine. The medicine program at UNSW is divided into three phases. Students in Phase 1 (Years 1 and 2) of the program engage in scenario-based learning, predominantly on the university campus, with a component of clinical and communication skills from the outset. Phase 2 (Years 3 and 4) is balanced between clinical attachments and campus-based learning of associated biomedical sciences, while students in Phase 3 (Years 5 and 6) are engaged in hospital- or community-based clinical attachments, integrated with a novel biomedical sciences curriculum supported by e-learning modules.

According to a needs analysis survey, the topics in Pathology that were most conceptually difficult for senior medical students were glomerulonephritis, lymphoma and leukaemia [3]. e-learning modules which bring together concepts in a clinical context are potentially effective ways of overcoming such perceived difficulty. We have previously shown that provision of e-learning modules on glomerulonephritis and lymphoma had a positive impact on learning in randomised controlled trials, compared with traditional teaching methods [3,9].

However, using PubMed and Google Scholar, we found no published reports that evaluated e-learning modules to assist medical students’ understanding of leukaemia. Therefore, this study aimed to evaluate the learning impact on senior medical students of a targeted e-learning module on leukaemia, compared with existing e-learning resources.

## Methods

### Development of an e-learning module on leukaemia

#### Authoring program

Adobe Captivate™ v5.5 was chosen as the authoring program because of its balance of design options and ease of use. Moreover, the published output can be displayed via any web browser, and the product can also be adapted to display on mobile devices (e.g. smart phones), in keeping with the current e-learning trend toward greater use of mobile technology [16].

#### Module design

A range of key criteria were utilised in the design of the e-learning module on leukaemia. The most important factors contributing to the efficacy of previous e-learning modules include [7,9,11,12]:

□ Authenticity - case based to optimise real-world relevance;

□ Interactivity - responds to users input, as well as being malleable for different types of learners and differing levels of ability;

□ Feedback - facilitates remediation of misconceptions; and

□ Integration - provision of an overarching conceptual framework that takes into account prior learning and curriculum objectives, and brings together concepts in a clinical context.

#### Framework

The aim of the module was to link the clinical approach to leukaemia with the basic sciences, particularly in relation to diagnostic protocols. This has been successfully achieved in previous UNSW pathology modules on other topics [9,17].

**Figure 1:**
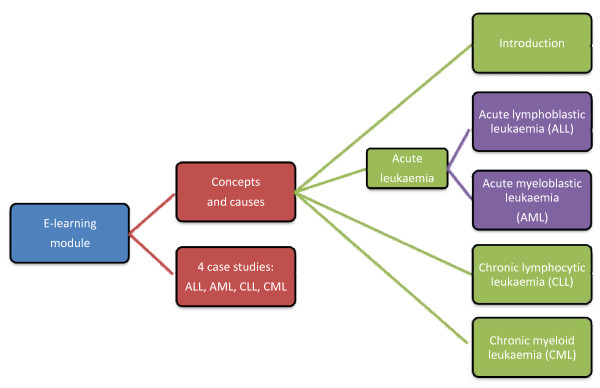
Schematic of the framework of the e-learning module on leukaemia.

Learning objectives were emphasised in the introductory screen of the module, which was divided into two main sections: ‘concepts and causes’ and ‘case studies’ (Figure 1). The concepts and causes section commenced with an introduction to leukaemia (definition, the function of normal peripheral blood leucocytes, and an analysis of cell lineages). Following the introduction there were three subsections, which dealt with the major concepts in pathogenesis and diagnosis of distinct disease profiles: acute leukaemia, including acute lymphoblastic leukaemia (ALL) and acute myeloid leukaemia (AML); chronic lymphocytic leukaemia (CLL); and chronic myeloid leukaemia (CML). The ‘acute leukaemia’ section dealt with both AML and ALL in a side-by-side comparison. Throughout the concepts and causes section, interactive tasks linked to learning objectives were included to enhance student engagement.

The concepts and causes section was intended to facilitate understanding of normal haematopoiesis and to identify abnormalities associated with leukaemia. Each of the characteristic disease profiles was discussed using the following structure:

1. Definition;

2. Epidemiology and risk factors;

3. Clinical features;

4. Diagnosis;

5. Staging and prognostic factors.

Four case studies were provided, each extracted from authentic cases of ALL, AML, CLL and CML respectively (these were generously provided by Prof. Fred Dee, University of Iowa). All case studies provided the de-identified patient’s history, relevant findings on examination, and results of diagnostic investigations. As details of each case were revealed on each successive screen, formative assessment questions and relevant feedback were presented regarding differential diagnosis, selection and interpretation of diagnostic investigations, and prognosis.

#### Interface and navigation

The interface was kept simple, uncluttered and consistent throughout the module, from the introductory pages to the case studies and glossary to prevent distractions and excessive cognitive load that might occur with a complex or unintuitive interface. Text on each screen was kept to a minimum to avoid overwhelming users by creating an on-line textbook environment. Audio was employed to supplement and expand on information provided in text, both to diversify the mode of presentation and to maintain engagement.

Perpetual functions included forward and back options, and a return to main menu button. Occasionally, a side branch from the main content path was utilised to provide additional or background information on a specific concept, for example, explaining the significance of immunoglobulin light chain monoclonality in CLL. These side branches were kept to a maximum of two screens, and subsequently returned the user to the screen from which they diverged.

#### Feedback and interactive features

**Figure 2:**
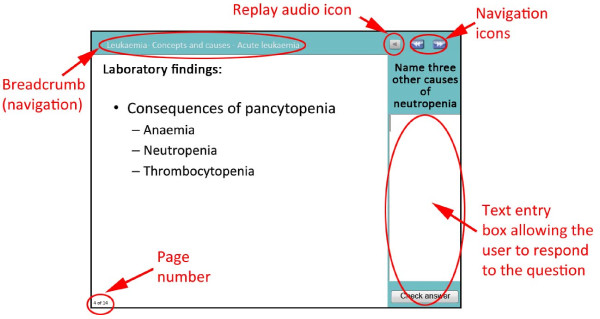
Annotated screenshot of an interactive question screen in the leukaemia module.

**Figure 3:**
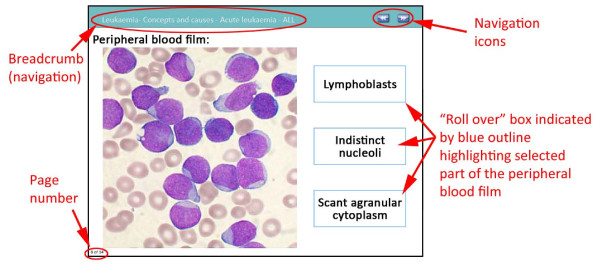
Annotated screenshot of an interactive slide in the leukaemia module.

In the context of e-learning modules, interactivity and feedback are core features in promoting learning, and immediate feedback is vital for the learning process [18]. Throughout the module, users are encouraged to interact with the concepts presented by answering questions and identifying features on images. Feedback on answers to the questions is provided immediately via audio or by a ‘roll over’ link (Figures 2 and 3). When the user holds the mouse cursor over a highlighted area on the screen (a roll-over) a text box appears with the answer. Additionally, some screens do not permit progress unless the question is attempted and feedback is returned, thereby preventing students from simply clicking through the module without engaging with the content.

Definitions of key terms were provided in a similar manner. Students can also directly access definitions from the screen containing the term through roll over boxes. The immediacy of access to information and feedback reduced the germane load (cognitive load required to process the interface) placed on students, allowing them to focus on the concepts presented [19].

#### Digital videos

Digital videos were embedded in the module to provide students with information about the diagnosis of common haematological disorders and to portray investigative procedures that they might not have the opportunity to witness during their clinical attachments.

#### Web access

The World-Wide Web was used as a means of distribution to geographically dispersed participants. The module was converted to a Flash™ file then uploaded to a web server, enabling users to view the module from home, at urban and rural clinical schools, as well as at the main university campus. Access was password-protected for the purposes of this trial. The module is available to view at http://web.med.unsw.edu.au/Pathology/Leukaemia/Leukaemia.htm.

### Study Design

#### Participants

Students enrolled in Phase Three (years 5 and 6, a cohort of 520 students) of the six-year undergraduate medicine program at UNSW in 2011 were invited via email to participate in a randomised controlled trial of the e-learning module on leukaemia, of which 45 responded (37 females, 8 males). These students were matched for academic ability (based on weighted average mark - ‘WAM’ - in the program) and gender (to minimise any gender-related differences). Volunteers were then randomised into either: the Study group (n = 23), who were provided with access to the new e-learning module; or the Control group (n = 22), who were provided with access to existing online resources. Participants were advised that their individual results from the pre-test and post-test would remain confidential and would not impact on their academic standing. The study received ethics approval from the UNSW Faculty of Medicine Human Research Ethics Advisory Panel (Ethics Approval No: 10095).

#### Instructions to participants

Participants were emailed instructions for the 2-week study period. The Study group email included a link to the module, with each individual receiving a unique username and password for the duration of the study, which they were asked to keep confidential. The control group was provided with links and encouraged to utilise currently available e-learning resources on leukaemia:

1. Robbins Pathologic Basis of Disease (8^th^ Edition) via MD Consult (available via the UNSW Library) - Section on Neoplastic Proliferations of White Cells; and

2. American Society of Hematology (ASH) Teaching Cases URL: http://teachingcases.hematology.org/

These resources were selected as equivalents to the ‘causes and concepts’ and ‘case studies’ components of our module, respectively. Importantly, the online textbook chapter addressed the causes and pathophysiology of leukaemia at the same (or greater) depth compared with our e-learning module. Further, the ASH cases titled ‘Childhood Acute Leukemia’, ‘A Patient with Pancytopenia’, ‘Lymphocytosis’ and ‘Myeloproliferative Disorder’ address CLL, AML, CLL and CML respectively at the same (or greater) depth compared with the cases in our e-learning module.

#### Pre-test, post-test and questionnaire

The pre-test, post-test and evaluation questionnaire were all designed using Questionmark Perception^TM^ (Questionmark, UK), a well-established suite of software for authoring and delivering web-based assessments and surveys. Feedback was provided for all questions upon completion of the pre-test and post-test.

The pre-test and post-test were administered immediately preceding and immediately following the two-week trial period, respectively. Both tests were based around case studies, to reflect the clinically oriented learning of senior medical students. Each case study included a clinical history, followed by several objective items. These were provided in several formats including standard multiple choice (single best answer), multiple response, and drag-and-drop (for image-based questions). Students were presented with haematology and immunohistochemistry results, and histological and radiological images, as appropriate for each case, then asked to answer questions relating to differential diagnosis, diagnostic investigations, provisional diagnosis, pathogenesis, and prognosis. Both the pre-test and post-test were reviewed by two senior members of the academic staff in Pathology at UNSW, as well as a senior clinical haematologist. All of them rated each test as being of equivalent difficulty, and ensured that the material covered by the test was addressed by both the e-learning module and the alternative e-learning resources. The post-test was authored after the e-learning module had been developed, thereby avoiding the potential bias of ‘teaching to the test’.

In addition, all participants were asked to complete a linked questionnaire at the conclusion of the post-test. The questionnaire obtained evaluative feedback regarding the module (Study group) and the alternative e-learning resources (Control group). Five-point Likert scales (1 = strongly disagree, 5 = strongly agree) were used for questions regarding module design and content. Free text responses were utilised to gather information about the most valuable features of the module, as well as suggestions for improvement. A PDF version of the questionnaire is available upon request.

### Evaluating the efficacy of the e-learning module

#### Statistical power

Prior to commencement of the study, it was determined that in order to show a 20 % difference between groups with statistical power > 99 %, a sample size of 15 participants per group was required (n = 30).

#### Quantitative analysis

All statistical analyses were performed using IBM SPSS Statistics™, version 19. Student’s t tests were performed to compare WAM, pre-test and post-test scores between groups. Stepwise linear regression analysis was performed to determine the factors that contributed significantly to variance in post-test scores. All data regarding WAM, pre-tests and post-tests for both groups are expressed as mean percentage scores ± standard error of the mean.

Data obtained from the evaluation questionnaires was analysed as follows: Kruskal-Wallis test and Dunn’s multiple comparisons tests were employed to compare participants’ ratings of the perceived difficulty of the topic of leukaemia before and after the trial, both within groups and between groups. Mann-Whitney U tests were performed to compare Likert scale data between groups. Likert scale and perceived difficulty data are expressed as median ratings ± interquartile range.

#### Qualitative analysis

Online evaluation questionnaires were administered immediately following the post-test to gather participants views regarding the e-learning module (Study group) and the alternative online learning resources (Control group). Open-ended questions were analysed for each group.

## Results

Of the 23 students in the Study group, 21 (91.3 %) completed all components of the trial, i.e., pre-test, post-test and questionnaire, while 21 of the 22 students in the Control group completed all components (95.5 %). There was no significant difference in the mean documented previous academic performance (WAM) for participants in the Study and Control groups, which were essentially identical (Study: 71 ± 1; Control: 71 ± 1) (t (43) = −0.163, P = 0.871).

**Figure 4:**
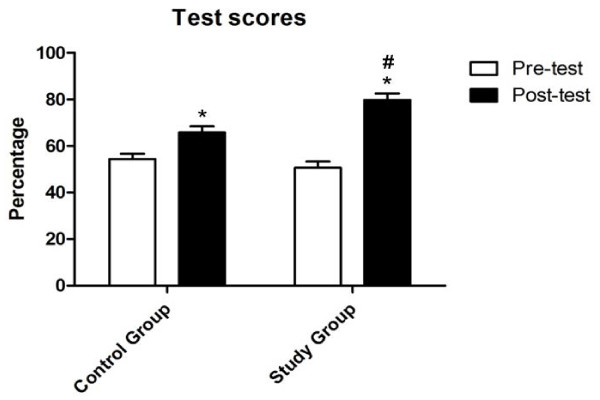
**Comparison of pre-test and post-test scores (mean and SEM) for participants in the Control and Study groups.** *Mean post-test scores greater than mean pre-test scores for each group (p < 0.05, t-test). #Study group mean post-test scores greater than Control group mean post-test scores (P < 0.001, t-test)**.**

There was no significant difference in mean percentage scores between groups for the pre-test on leukaemia (Study: 51 ± 3; Control: 54 ± 2) (t (43) = 1.055, P = 0.297). However, the Study group achieved significantly higher mean percentage scores in the post-test on leukaemia (Study: 80 ± 3; Control: 66 ± 3) (t (42) = −3.591, P < 0.001). Importantly, this striking difference was noted even though the Control group’s performance also improved significantly compared with the pre-test (Figure 4).

The Study group reported spending more time on average studying the topic of leukaemia than the Control group during the 2-week trial period, but this difference (equating to three minutes per day) was not statistically significant (Study: 2.3 ± 0.4 hours; Control: 1.6 ± 0.4 hours) (t (39) = −1.347, P = 0.186).

In a stepwise multiple regression analysis, group allocation was the only significant predictor of performance in the post-test (R = 0.516, R ^2^ = 0.266, P = 0.001), i.e. 26.6 % of the variance in post-test scores was accounted for by group membership. In this model, neither known academic ability nor time spent studying was predictive, although the latter parameter in isolation correlated significantly with post-test scores (R = 0.363, P = 0.02). The correlation between WAM and post-test scores was not statistically significant (R = 0.294, P = 0.052).

**Figure 5:**
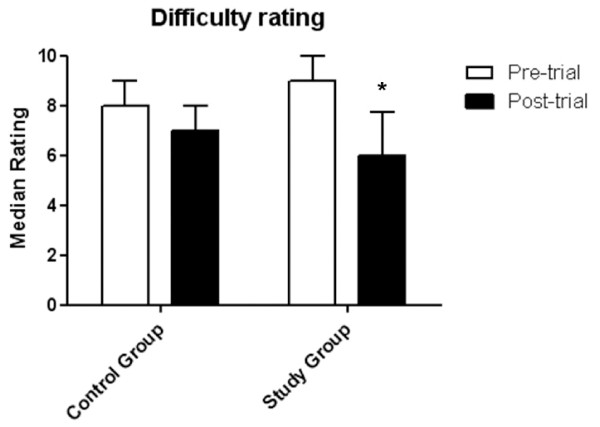
**Comparison of pre-trial and post-trial ratings (median ± interquartile range) of the difficulty of leukaemia by participants (1 = least difficult, 10 = most difficult) between Control and Study groups.** The Study group’s post-trial perceived level of difficulty was significantly lower than pre-trial (* P < 0.001, Kruskal Wallis ANOVA followed by Dunn’s multiple comparisons test)**.**

Via the online evaluation questionnaire, participants in both the Study and Control groups rated the perceived difficulty of the topic of leukaemia before and after the trial period on a 10-point Likert scale (1 = least difficult, 10 = most difficult). Pre-trial, there was no significant difference between groups in ratings of the perceived difficulty of leukaemia (Study median rating: 9; Control median rating: 8). Post-trial, there was a significant decrease in the median perceived difficulty of leukaemia in the Study group (median rating pre-trial 9; post-trial 6, P < 0.001, Dunn’s multiple comparisons test), but not in the Control group (median rating pre-trial 8; post-trial 7) (Figure 5).

**Figure 6:**
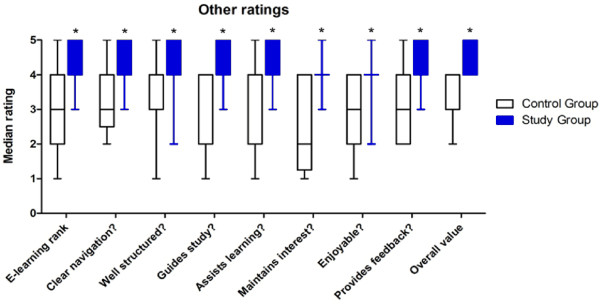
**Comparisons of Likert scale questionnaire responses from Control and Study groups, relating to their perceptions of existing e-learning resources and the e-learning module on leukaemia respectively.** Data are represented by median ± interquartile range (1 = strongly disagree, 5 = strongly agree). The Study group rated all aspects significantly higher than the Control group (* P < 0.001, Mann-Whitney U test)**.**

The Study group’s online evaluations of the e-learning module were overwhelmingly positive, and their rating of each aspect (including enjoyment, guide to study and overall value for learning) was significantly higher compared with evaluations of the alternative e-learning resources by the Control group (all P < 0.001, Mann-Whitney U tests) (Figure 6). Of particular note, the Study group found the module to be the most useful resource for learning about leukaemia, compared with lectures, tutorials, private study and clinical experience, whereas the median ranking of the e-learning resources available to the Control group was third out of five.

Students in the Study group found the following features of the module most helpful: interactivity; feedback; case studies; multimedia (videos, animations, audio); and histopathological images. In addition, participants in the Study group commented that revision of basic science concepts in the concepts and causes section of the module was extremely useful.

Below is a selection of representative comments from the participants in the Study group:

"“Accessible, easy to follow, provided key information.”"

"“Information was presented succinctly and was available for use in my own time, and as many times as required. It's good to have a guide on what we're expected to know, rather than being told to 'go learn leukaemia'.”"

"“The very nature of an e-learning module is much easier than having to sift through books. It is easy for me to get to, and I can do it whenever I feel like. Testing and feedback are crucial.”"

"“Best features: well structured, clear, easy to follow, teaching concepts and causes ,reinforced with cases where we were encouraged to think, recall and answer questions.”"

"“Interactivity helped maintain interest; the case studies were of a good digestible length. Overall, an enjoyable module that complemented my learning (and inspired me to do more reading)!”"

"“Interactive information and questions with immediate feedback so can correct misunderstanding straight away; simple navigation; ability to replay audio; multimedia (diagrams, videos, audio… particularly liked the diagram of haematological "family tree" with the interactive feature of fading out cells not involved in each condition).”"

"“Particularly liked the splenomegaly video and how the commentary linked back to the patient's presentation. The other linked videos were also very good (bone marrow biopsy video fantastic idea for those who haven't had the chance to see one in a clinical situation). Matching the interactive elements to the nature of the content (e.g. labels on pictures, matching lists of symptoms and pathophysiology etc) made it easy and fun to self-test and discover new info.”"

Study group participants also provided feedback to improve the module. Several suggested that a transcript of the audio should be available on each screen. This feature has subsequently been implemented. Participants generally preferred more text on each screen, rather than audio. Some participants, who had limited internet speeds due to their rural location, found that the module took a long time to download.

In contrast, online evaluation data provided by the Control group was less positive. The participants in the Control group indicated that the content of the e-learning resources they accessed was not sufficiently tailored to their context. Participants frequently commented that the information was too theory-based, yet they perceived that the pathophysiology underlying the results of diagnostic procedures was not well explained.

Control group participants suggested that the online learning resources needed to be more interactive, and more specific to their syllabus. Students commented that while there were “a lot of resources out there” many students have issues deciding “what level of knowledge is expected”.

Below is a selection of representative comments from the Control group:

"“Boring, not interactive, excessive detail.”"

"“The ASH resource was user friendly. However the case studies were not always labelled and the information related to an American context. I accessed the Robbins resource, however I did not use this as I find reading large segments of text online very difficult to concentrate on and remember.”"

"“I didn't like how it amalgamated leukaemia and lymphoma - although they are similar it only further added to my confusion between the two.”"

"“I found them hard to engage with (didn't really capture my attention).”"

"“Robbins text contained too much detail, teaching cases left out detail. Both approaches are off-putting.”"

## Discussion

As expected, the mean scores of both groups improved from pre-test to post-test, indicating that the existing online resources provided to the Control group did confer some benefit. However, the learning benefits of the tailored e-learning module utilised by the Study group markedly exceeded those of the resources provided to the Control group. Although the cohort size for this trial was relatively small, the statistical power of the study was sufficient to yield valid results.

Both the pre-test and post-test were reviewed by two senior members of the academic staff in Pathology at UNSW, as well as a senior clinical haematologist. All of them rated both tests as being of equivalent difficulty, and ensured that the material covered by the post-test was addressed by both the e-learning module and the alternative e-learning resources. Furthermore, the post-test was developed after the e-learning module had been completed, thereby avoiding the potential for ‘teaching to the test’. This reduces the likelihood of bias towards the Study group in the design of the post-test.

The Study group’s success might be at least partially accounted for by the targeted nature and overarching conceptual framework provided by our e-learning module. Certainly, the lack of such focus in the existing online resources was remarked upon by participants as a major disadvantage. Furthermore, in accordance with reports of previously successful e-learning interventions [7,9,11,12], the high level of interactivity and dialogicfeedback provided by the e-learning module, particularly compared with the online text book,is likely to have resulted in improved efficacy. In any form of e-learning, those features are important for both engagement and educational impact [11,15,20,21].

Surprisingly, stepwise regression analysis revealed that the variance in post-test scores was significantly influenced only by group allocation. It was anticipated that time spent studying the topic and students’ previous academic performance (WAM) would also have had significant effects. Indeed, of those variables, only time studying leukaemia correlated significantly with post-test scores, whereas the correlation between post-test scores and WAM was not statistically significant. From these data, we infer that the e-learning module provided significant learning gains, without requiring a significant extra investment of students’ time. It seems reasonable to conclude that the design of the module provided students with a time-efficient learning experience. The weak relationship between WAM and post-test scores might be accounted for by the benefits of e-learning for weaker performing students, more so than higher performers. This phenomenon is consistent with previous studies of e-learning interventions [22,23].

The questionnaire data revealed that the e-learning module significantly reduced the perceived difficulty of leukaemia for participants in the Study group at the end of the trial period, compared with the Control group. This is an important finding, which suggests that exposure to the integrated conceptual framework of the e-learning module had an impact not only on learning, but also on confidence and attitudes. This is reinforced by the significantly increased interest in leukaemia espoused by participants in the Study group compared with those in the Control group.

The majority of participants in the Study group rated the e-learning module as being the most helpful mode for their study of leukaemia (compared with lectures, tutorials, texts and individual study), while students in the Control group rated the existing e-learning resources as being significantly less helpful. Those in the Study group remarked that interactivity and feedback within the module added to their enjoyment of the topic. Participants also commented that their ability to understand and interpret the case studies was important to their success in the post-test. These data indicate that the e-learning module had both qualitative and quantitative beneficial effects on learning. These observations are consistent with previous studies of e-learning interventions [9,13,18].

The current study expands on earlier reports of studies regarding e-learning modules on lymphoma [9] and glomerulonephritis [2]. The validity of this trial was also bolstered by the inclusion of a pre-test, which established that the mean baseline knowledge of leukaemia was equivalent for both the Control and Study groups.

Many studies have compared e-learning to face to face teaching in a wide variety of contexts. However, there have been few randomised controlled trials that have compared the learning impact of different forms of e-learning [10] and this is the first such report regarding the topic of leukaemia. Furthermore, we believe that this study has important implications for the design of future e-learning modules. In particular, the findings suggest that targeted e-learning modules which embed case-based examples within an overarching conceptual framework are much more effective than generic online cases and online texts. Indeed, our results highlight the notion that in the sphere of e-learning, not all formats are equal in value [24].

## Conclusions

The results of this randomised, controlled trial indicate that a purpose-built e-learning module on leukaemia significantly improved students’ understanding of the topic, and measurably decreased students’ perceptions of its difficulty. The e-learning module on leukaemia had a significant impact on learning in this cohort, compared with existing online resources. We believe that the curriculum-based conceptual framework embedded in the structure of the module, as well as interactivity and dialogic feedback, contributed to its impact. The results of this study have implications for the design of e-learning in medicine and other disciplines.

## Competing interests

The authors declared that they have no competing interests.

## Authors' contributions

YM was involved in the design, development and implementation of the e-learning module on leukaemia described in this study, as well as drafting the text of this article. RL supported the development and implementation of the e-learning module on leukaemia, as well as editing the text of this article. RKK supported the design, development and implementation of the e-learning module on leukaemia, as well as assisting with data analysis and editing the text of this article. GMV is guarantor and was involved in the design, development and implementation of the e-learning module on leukaemia. He was also responsible for the study design and data collection, as well as assisting with data analysis and editing the text of this article. All authors read and approved the final manuscript.

## Pre-publication history

The pre-publication history for this paper can be accessed here:

http://www.biomedcentral.com/1472-6920/12/36/prepub
